# Hepatic Glucagonoma in a Post-bariatric Female Patient: A Case Report

**DOI:** 10.7759/cureus.46724

**Published:** 2023-10-09

**Authors:** Sophia Garcia, Saly J Canela Ynoa, Arturo M Concepción, Flávio A De Sá Ribeiro

**Affiliations:** 1 General Surgery, Instituto de Pós Graduação Médica Carlos Chagas, Rio de Janeiro, BRA; 2 General Surgery, Instituto de Pós Graduacão Médica Carlos Chagas, Rio de Janeiro, BRA

**Keywords:** glucogonoma, necrolytic migratory erythema, diabetes mellitus, general surgery, neuroendocrine tumor

## Abstract

Glucagonomas, neuroendocrine tumors originating from the pancreas marked by excessive glucagon secretion, present a diagnostic challenge due to their rarity and diverse symptomatology. In this report, we present a 47-year-old female with a history of bariatric surgery, diabetes mellitus, and deep vein thrombosis who exhibited weight loss, anemia, migratory necrolytic erythema on the lower limbs and groin, and fecal incontinence. Imaging revealed liver secondary lesions without an identifiable primary tumor. After undergoing surgery, a pathologic examination of the excised tissue confirmed that the lesions were a glucagonoma. This case underscores the imperative of how common side effects of bariatric surgery could mask symptoms, delaying the diagnosis of glucagonomas.

## Introduction

Glucagonomas are rare glucagon-secreting neuroendocrine tumors (NETs) originating from the alpha cells of the pancreas [1]. They are typically large (greater than 3 cm) and mainly located in the tail or body of the pancreas [2]. The incidence rate of glucagonomas is approximately 2.4 per 100,000,000 in America and 2.6 per 100,000,000 in Japan [3]. Over 50% are metastatic at the time of diagnosis, with similar incidence rates in males and females, and most patients with glucagonomas present in the fifth to sixth decade of life [2]. These NETs secrete glucagon, causing a combination of symptoms known as glucagonoma syndrome. These symptoms include the skin disorder necrolytic migratory erythema (NME), which occurs in approximately 90% of cases [2,3]. This characteristic rash is diagnosed both clinically and histologically. Clinically, it presents as a widespread painful, scaly erythematous patch, with the main areas of involvement being the perioral, perigenital regions, and the extremities. Histologically, it is characterized by parakeratosis with the loss of the granular layer, necrosis, and separation of the upper epidermis, along with vacuolization of the keratinocytes, dyskeratotic keratinocytes, and neutrophils in the upper epidermis [2,4]. Other associated symptoms include diabetes mellitus, stomatitis, weight loss, anemia, glossitis, cheilitis, steatorrhea, and diarrhea [1,3]. Additionally, the clinical course can be complicated by venous thrombosis and pulmonary embolism (occurring in 30 to 50% of cases), as well as neuropsychiatric disturbances, including depression, psychosis, agitation, dementia, paranoid delusions, ataxia, hyperreflexia, and optic atrophy. Due to a high risk of recurrence, follow-up is necessary [1].

Glucagonomas should be suspected in patients with NME, whether associated with other symptoms or not. Elevated serum glucagon levels, over 500 pg/mL, along with serum concentrations of amino acids and zinc, should be assessed. A complete blood count and blood smear should be obtained to check for concurrent normocytic anemia. It is also essential to get serum levels of parathyroid hormone, gastrin, insulin, pancreatic polypeptide, serotonin, vasoactive intestinal polypeptide, prolactin, and adrenocorticotropic hormone, as glucagonomas can rarely be associated with multiple endocrine neoplasia type 1 (MEN1) syndrome. A helical multiphasic contrast-enhanced CT scan is recommended, while MRI should be performed in cases of indeterminate lesions and may have better sensitivity in detecting liver metastases [2,4]. The objective of this case report is to present a 47-year-old female patient who, following bariatric surgery, was diagnosed with glucagonoma, with no apparent primary tumors identified in her pancreas.

## Case presentation

In February 2023, a 47-year-old female patient went to a general surgeon's office, with complaints of general discomfort, anemia, erythema on the lower body, and diarrhea. Given her fragility and concerning symptoms, she was admitted to a hospital in Rio de Janeiro, Brazil.

She had a surgical history of a gastric bypass in August 2022 and also a five-year progression of diabetes mellitus, hypothyroidism, and a history of deep vein thrombosis (DVT) in October 2022.

During her pre-operative checkup for the bariatric surgery, a CT scan showed some hepatic nodules, which were presumed to be hemangiomas. Nevertheless, after the bariatric surgery, she had a worsening of her general status and showed refractory anemia, along with sudden weight loss and erythema on her legs and groin, with fecal and urinary incontinence. As months passed, the symptoms worsened, prompting her to get a medical assessment.

**Figure 1:**
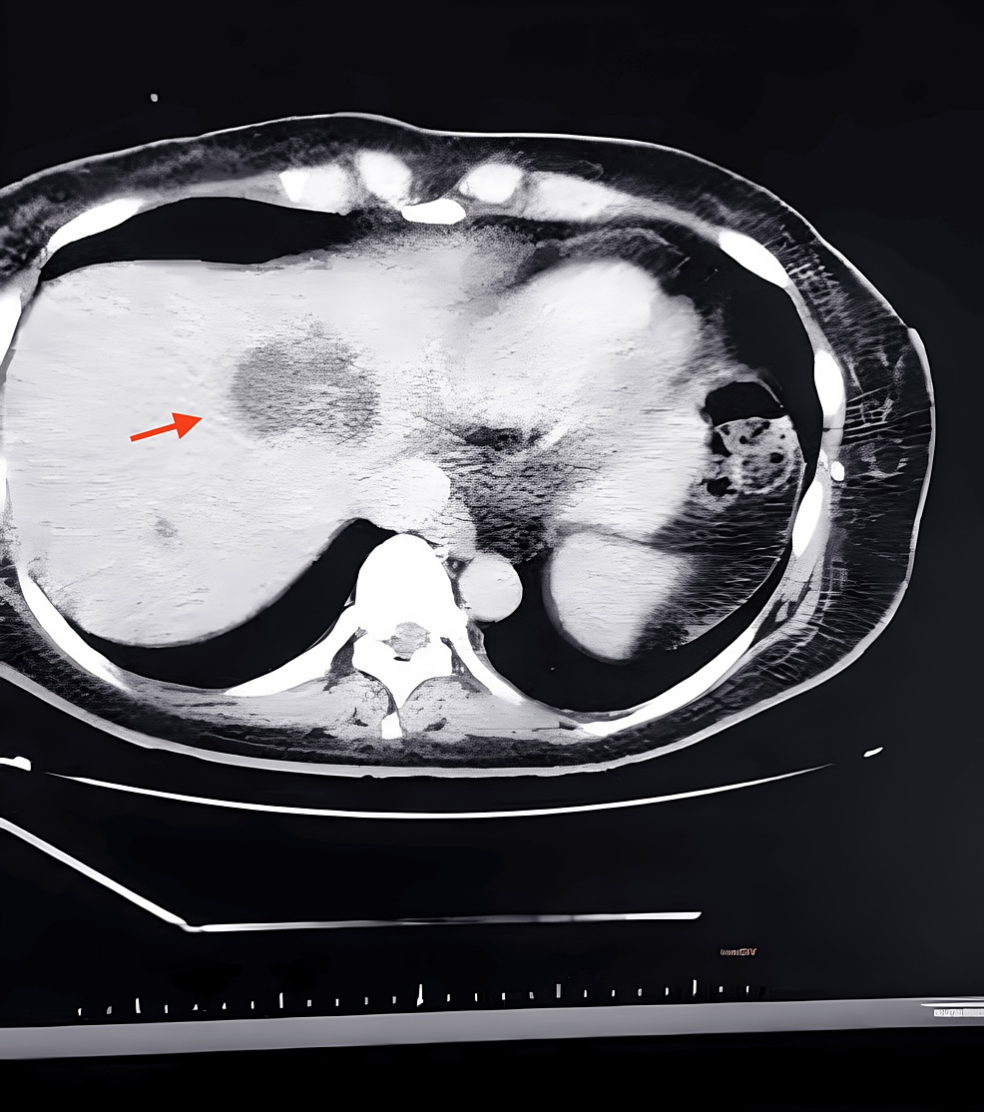
CT at Admission The red arrow shows the main hepatic lesion.

Upon admission to the hospital, a CT was performed, revealing secondary neoplastic lesions on her liver, with no identifiable primary tissue source (Figure 1). The main findings from the tomography report indicated an expansive, heterogeneous lesion, measuring 7.8 x 5.4 cm, affecting hepatic segments II and IV-A, with areas of cystic/necrotic degeneration. Additionally, there were several smaller lesions scattered throughout the parenchyma, strongly suggestive of secondary neoplastic involvement.

**Figure 2:**
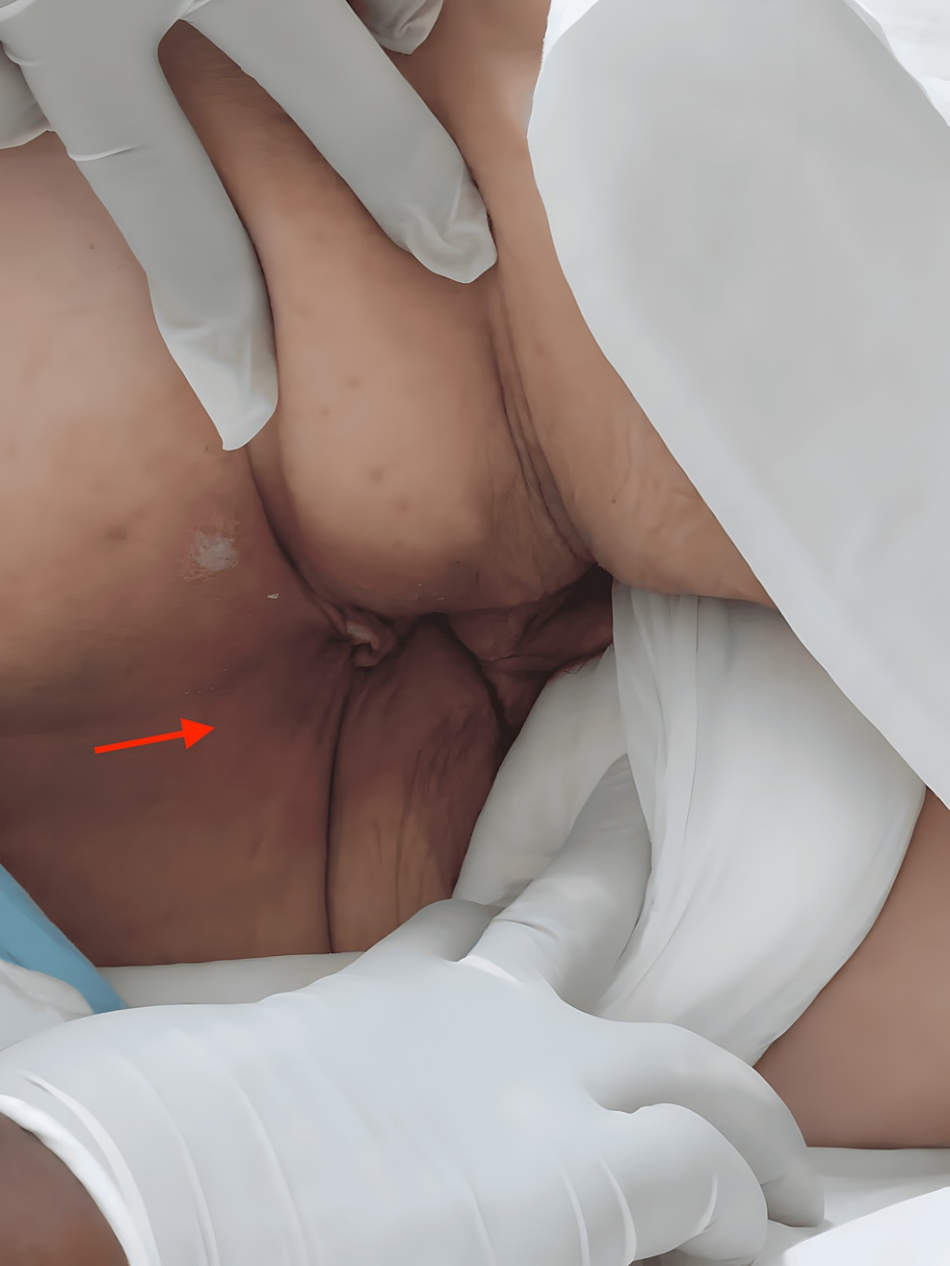
Patient's Migratory Necrolytic Erythema The picture shows the groin region of the patient; the red arrow highlights where erythema is evident.

At admission, the physical examination revealed a subdued mood and a flaccid abdomen, painful upon deep palpation, and both inferior limbs had erythematous lesions extending up to the groin region, with no signs of DVT (Figure 2).

Laboratory measurements upon admission are shown in Table 1.

**Table 1:** Laboratory Findings Upon Admission

Laboratory test	Value	Reference Range
Hemoglobin	6.6 g/dL ¯	12.0-16.0 g/dL
Hematocrit	22.3% ¯	35.0-46.0%
Leukocytes	3.31/µL ¯	4.0-11.0/µL
Platelet count	214,000 /µL	150,000-450,000/µL
Glucose	94 mg/dL	70-99 mg/dL
Urea	16 mg/dL	10.0-50.0 mg/dL
Creatinine	0.5 mg/dL¯	0.6-1.3 mg/dL
Sodium	139 mEq/L	136-145 mEq/L
Potassium	3.1 mEq/L ¯	3.5-5.1 mEq/L
Calcium	7.8 mg/dL ¯	8.5-10.4 mg/dL
Magnesium	1.8 mg/dL	1.7-2.3 mg/dL
Prothrombin time	25.7 secs ­	11-13.5 secs
Lactic acid	0.9 mmol/L	0.4-2.0 mmol/L

Based on the patient's medical history, imaging findings, and physical examination, a diagnosis of a NET associated with a glucagonoma was considered. As part of the treatment plan, the patient received a transfusion of three packs (500 ml each) of red blood cell concentrate, and octreotide was started. Additionally, the administration of Clexane (enoxaparin) was suspended, and fasting measurements of glucagon, insulin, and glucose were solicited. Surgery was scheduled for the following day as the next step in the patient's management.

**Figure 3:**
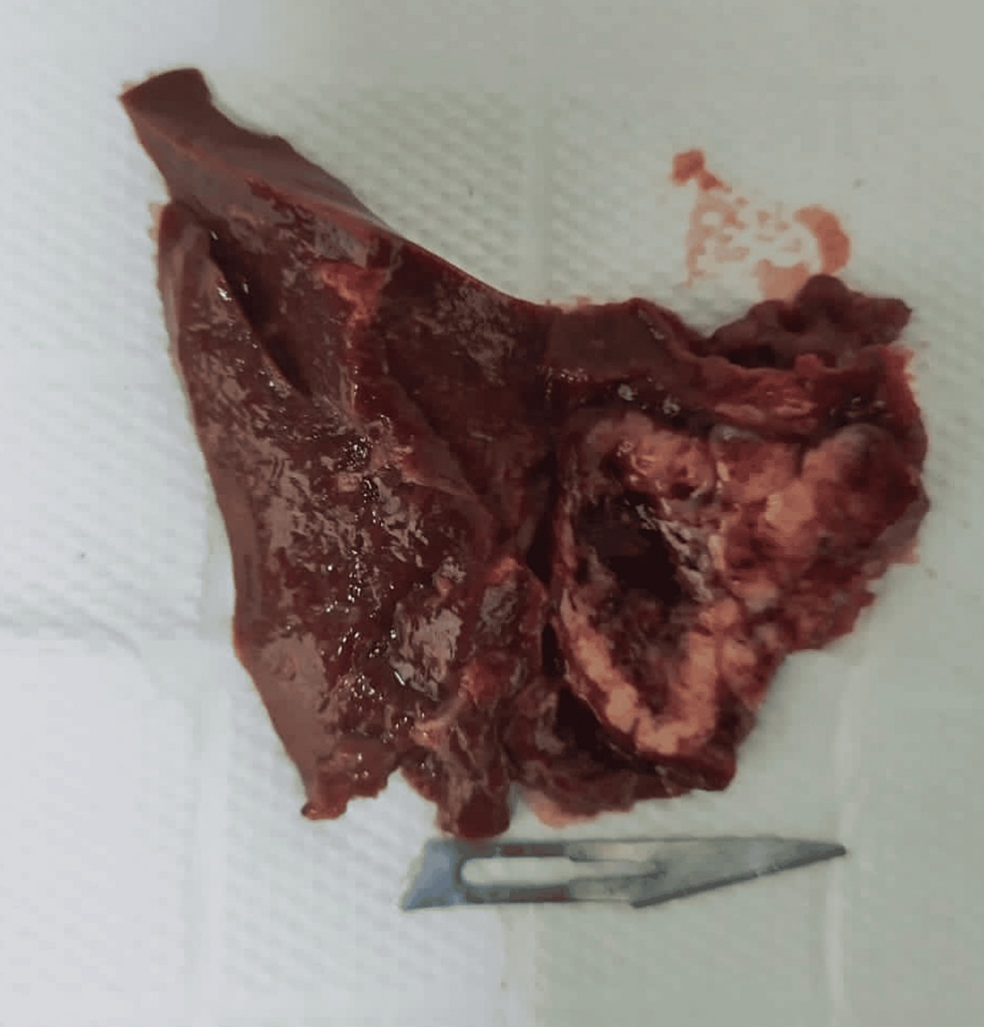
Excised Tissue Segment IVa of the liver

**Figure 4:**
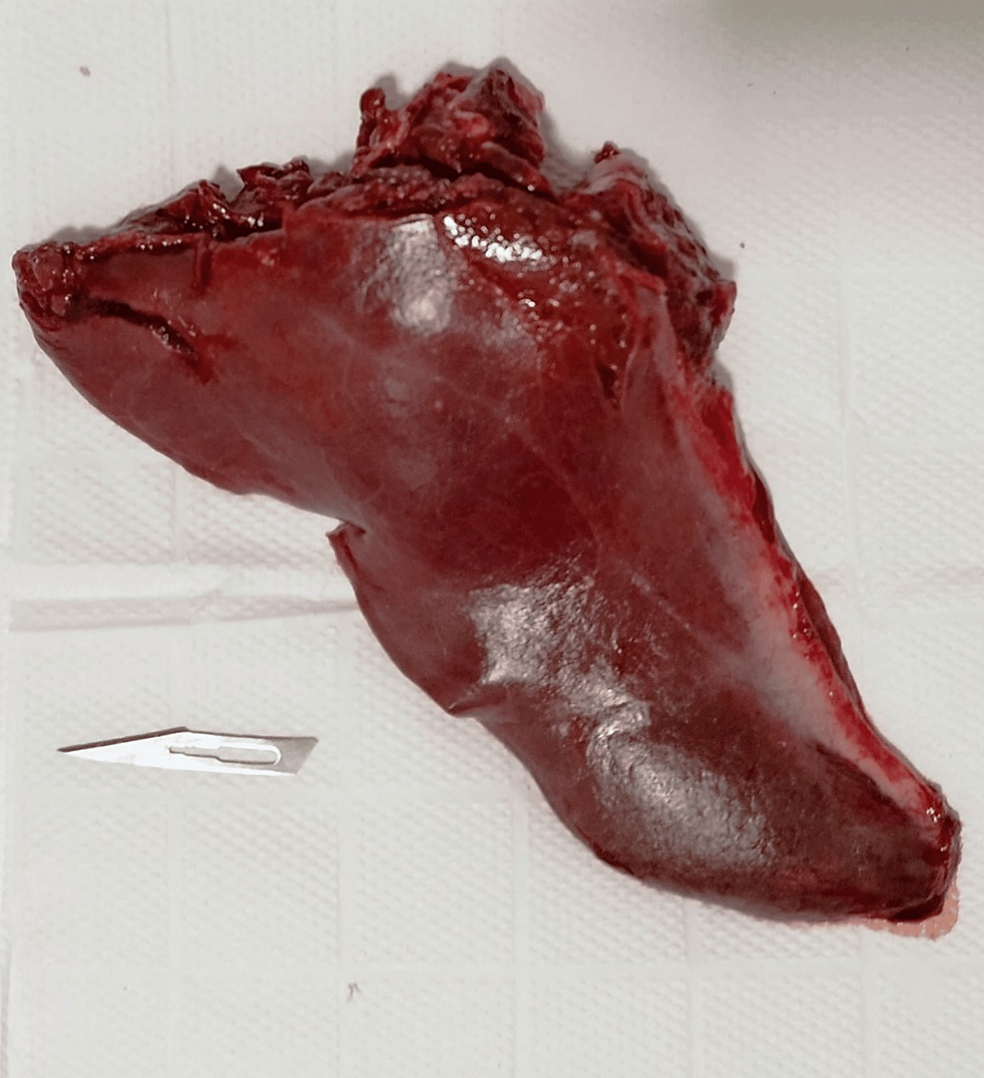
Excised Tissue Segments II and III of the liver

**Figure 5:**
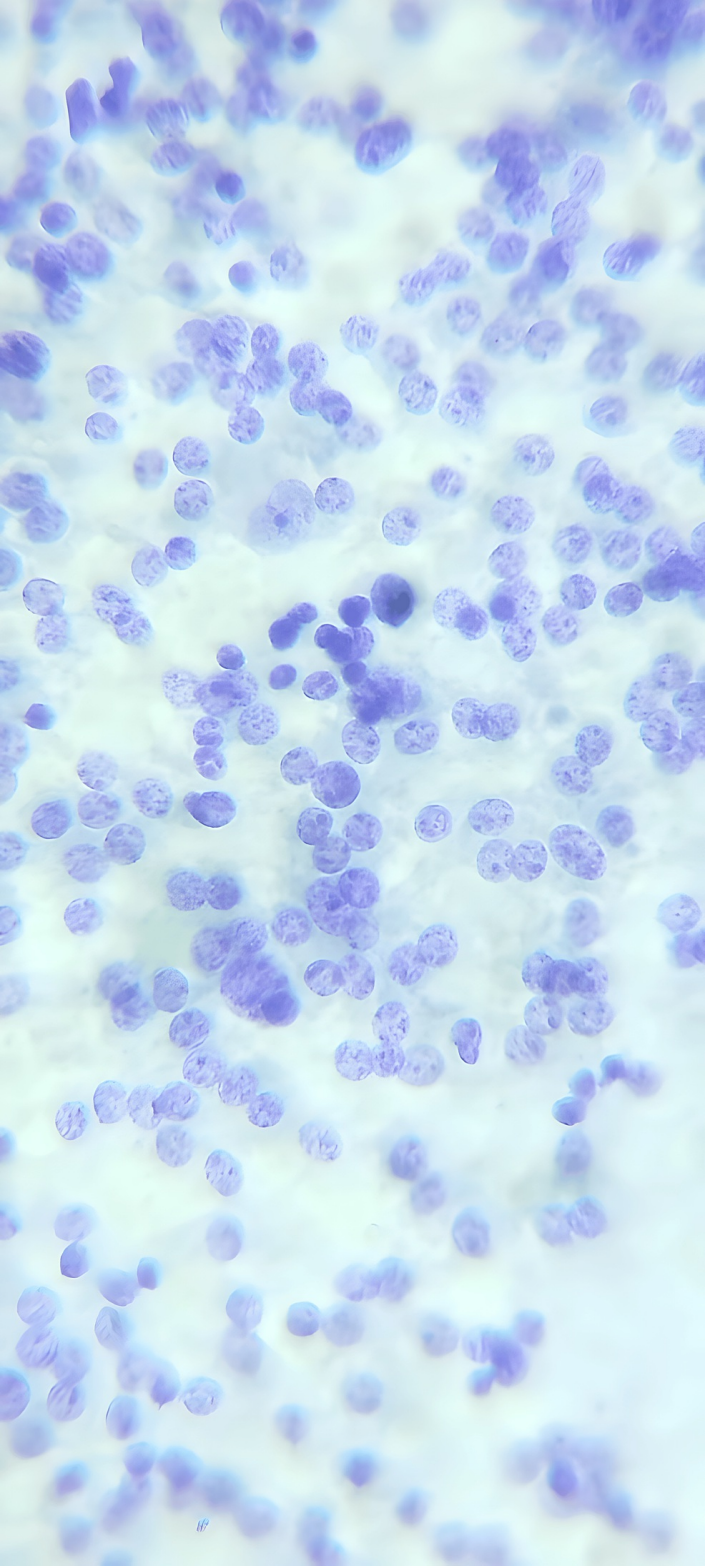
Microscopy of the Excised Tissue The image shows a neuroendocrine tumor

Following the initial surgical plan, the patient underwent surgery the following day. Unfortunately, due to a lack of hospital resources, measurements of glucagon and insulin were not obtained as initially intended. During the surgical procedure, which involved video-laparoscopic hepatic biopsy, a pathologist was present to collect a tissue sample from the surgical site (Figures 3, 4). This tissue sample was subsequently subjected to cryosection analysis, revealing the presence of a NET. Afterward, we opted for partial hepatectomy of segments II, III, and IVa to retire the lesions and alleviate the symptoms. Further confirmatory testing identified this tumor as a glucagonoma (Figure 5). The surgery itself was successful, and the patient was transferred to the intensive care unit for post-operative care. To further support her recovery, amoxicillin and clavulanate were added to her prescription.

**Figure 6:**
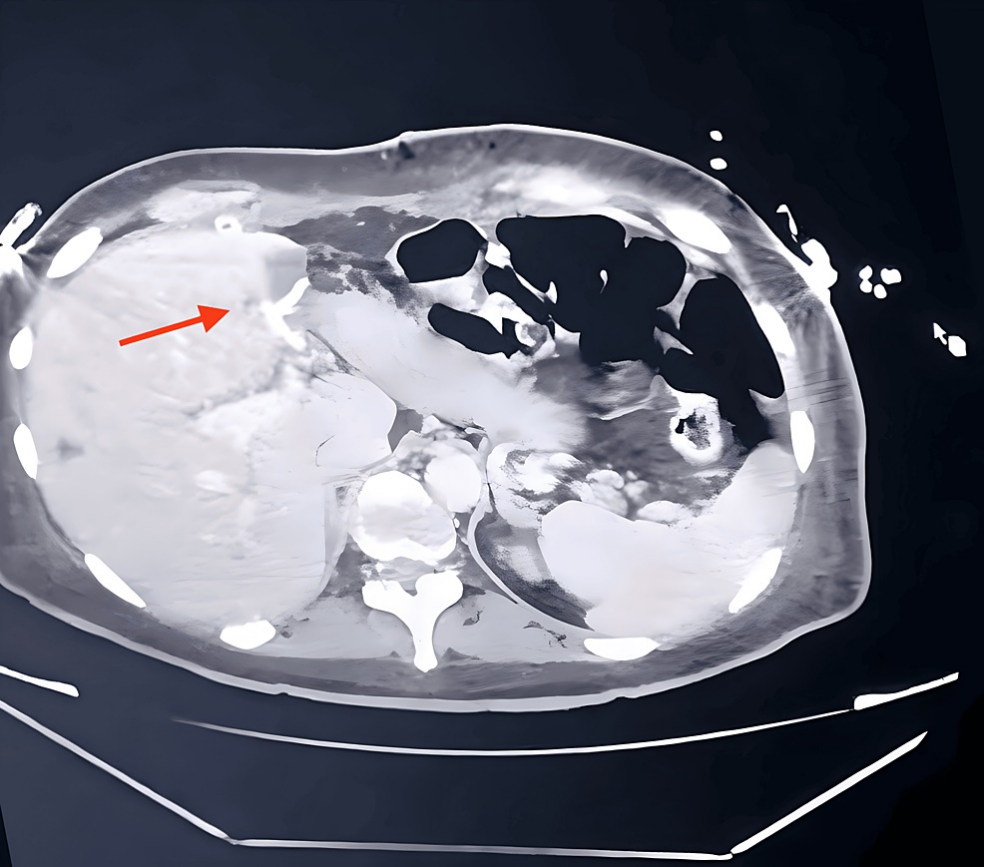
Post-surgical Computed Tomography The red arrow shows segment IVb

Subsequently, a post-surgical computed tomography was conducted, revealing several notable findings. These included the insertion of a drain on the right flank with the endpoint on the left flank, evidence of partial hepatectomy of the lateral segments of the left lobe, and indications of partial resection of the largest suspected liver lesion, previously measured at 7.5 x 5.5 cm but now measuring 5.2 x 4.7 cm in segment IV-a. Furthermore, a new heterogeneous elongated lesion with contrast medium impregnation was identified in segment IV-b, measuring 3.0 x 2.3 cm, adjacent to the medial hepatic border and the surgical clips (Figure 6).

The remainder of the liver displayed a regular contour with multiple hypodense nodular lesions, the largest of which exhibited heterogeneity, areas of cystic/necrotic degeneration, and irregular contrast enhancement, raising suspicions of secondary lesions. The patient remained in the hospital for 10 days following her surgery, during which she received two additional blood transfusions. Subsequently, she was discharged and placed under ongoing supervision to monitor her recovery and progress. As of now, eight months after surgery, the patient is in a stable clinical condition and being treated for malnourishment.

## Discussion

Glucagonomas are excessively rare tumors that are not easily diagnosed. According to an epidemiological study, the average time between the occurrence of symptoms and the actual diagnosis was two and a half years, and of the diagnosed patients, half of them already had tumor metastases, and about 82% of them presented with NME, demonstrating that this clinical sign can be determinant on glucagonoma diagnosis [3]. In our study, we present a patient who exhibited many glucagonoma symptoms (diabetes, anemia, diarrhea, DVT, decreased mood). The patient's history of gastroplasty is a noteworthy aspect of this case. Bariatric surgery, designed to promote weight loss, led to a significant reduction in the patient's body weight. This weight loss, coupled with nutritional deficiencies, masked the underlying glucagonoma, delaying its diagnosis. It is essential to recognize that the symptoms associated with glucagonoma, such as weight loss and malnutrition, may be attributed to the effects of bariatric surgery alone and not correlated to another underlying disease. Moreover, it is worth noting that zinc deficiency is a common manifestation of malnutrition in the context of glucagonoma and should always be assessed.

With an estimated incidence of one in two million people and unclear pathogenesis, some glucagonomas are associated with mutations in the MEN1 gene. At the same time, malnutrition, particularly deficiencies in zinc, amino acids, and fatty acids and liver dysfunction have been hypothesized as potential causes of this rare tumor. Differential diagnosis is further complicated by the existence of an even rarer syndrome known as pseudo glucagonoma syndrome. Additionally, cutaneous manifestations of other disorders can introduce diagnostic challenges due to their similarity to NME, a characteristic sign of glucagonoma. Examples of such disorders include pellagra, eczema, acrodermatitis enteropathica, pemphigus, and candida [5].

Interestingly, there is no clear correlation between the NME and the presence of hyperglucagonemia, as this sign can also be present in other diseases such as cirrhosis, malabsorption disorders, inflammatory bowel disease, and chronic pancreatitis. However, it is important to note that in some cases, this distinctive skin eruption may serve as the initial manifestation of the disease. Patients may initially receive topical treatments with antibiotics or antifungals, but these treatments mostly yield refractory results [6]. An example of this was presented in a glucagonoma case report that described a patient with NME who was treated with corticosteroids for two years without improvement. It was only after the tumor was diagnosed and surgically removed that the patient experienced a rapid relief of symptoms [7]. 

Several case reports have highlighted the diagnostic challenges and delays associated with glucagonoma, often leading to diagnostic confusion. One case report highlighted the incidental nature of diagnosis in a patient whose serum glucagon levels were nearly within the normal range (225 pg/mL). Typically, a glucagonoma diagnosis is considered with levels of 500 pg/mL or higher. In this unique case, the diagnosis became possible due to the fortuitous measurement of serum glucagon levels coinciding with the incidental discovery of a pancreatic mass [8]. Likewise, another case report documented cerebral sinus venous thrombosis as the initial presentation of a glucagonoma. This highlights that while DVT is the most common coagulopathy manifestation associated with glucagonoma, it is not the exclusive one. Clinicians should remain vigilant for other common features of glucagonoma, including diabetes, sarcopenia, metabolic deficiencies, and weight loss [9].

When a glucagonoma is suspected but cannot be identified via abdominal CT scan, selective celiac, and superior mesenteric arteriographies are preferred due to the tumor's pronounced hypervascularity. Diagnosis confirmation involves histopathological techniques, including immunohistochemistry, electron microscope analysis, and in situ hybridization of glucagon messenger RNA [6]. Surgical resection offers the only chance for a cure, but this applies to a minority of cases where the tumor is localized at diagnosis. In non-curable cases, somatostatin analogs like octreotide and lanreotide can mitigate the effects of glucagon excess and inhibit glucagon secretion. Additionally, antibiotics, zinc replacement, and steroids may improve NME. Nutritional support, including amino acid supplementation, can address malnutrition and catabolism resulting from glucagon excess. Prophylactic anticoagulant therapy, such as heparin, is advisable to prevent deep venous thrombosis during the perioperative period. Glucagonomas are typically slow-growing; however, they are often advanced at the time of diagnosis. Survival prognosis depends on factors like age, tumor grade, and the presence of distant metastases. Achieving a cure is rare once the tumor has metastasized [2].

In cases of hepatic metastasis, resection has been recommended in patients without widespread liver involvement, diffuse extrahepatic metastases, and decreased liver function. Resection has led to a decrease in glucagon levels and significant improvement in NME [2]. In this case, the diagnosis was confirmed intrasurgically, and the decision to excise the metastases was made to improve the patient's clinical condition. Even though the diagnosis was challenging and the chosen management was controverted, it resulted in improvement of the patient's clinical manifestations, only remaining with malnutrition that has been able to be treated at home.

Our case, to the best of our knowledge, is the only reported case report of a post-bariatric patient developing a glucagonoma. This case underscores the critical importance of considering all presenting symptoms, both typical and atypical when diagnosing a disease. Despite the patient's relatively young age compared to the typical age of presentation, all symptoms were evident. However, our study does have limitations, primarily the absence of certain laboratory measurements that could have further enriched the case. Our patient received nutritional support to mitigate the effects of the tumor, as complete excision, unfortunately, was not possible.

## Conclusions

In conclusion, this case report sheds light on the rare occurrence of hepatic glucagonoma in a post-bariatric female patient, a scenario previously unreported in medical literature. Glucagonomas are indeed challenging to diagnose due to their rarity and the diverse symptoms, which often lead to diagnostic delays. For this patient, some of these signs were masked by the effects of a bariatric surgery. Early recognition, multidisciplinary care, and thorough evaluation are the key to achieving optimal outcomes for patients. Further research and reporting of cases like this one will contribute to a better understanding of this rare NET and improve patient care and management strategies.
